# Pancreatic outflow tract reconstruction after pancreaticoduodenectomy: a meta-analysis of randomized controlled trials

**DOI:** 10.1186/s12957-021-02314-2

**Published:** 2021-07-06

**Authors:** Xin Xin Wang, Yu Ke Yan, Bao Long Dong, Yuan Li, Xiao Jun Yang

**Affiliations:** 1 Department of General Surgery, Gansu Provincial Hospital, No. 204 Donggang West Road, Lanzhou, 730000 Gansu Province China; 2 The 1st Clinical Medicine College, Gansu University of Chinese Medicine, Lanzhou, 730000 Gansu Province China; 3 Peoples Clinical Medicine College, Lanzhou University, Lanzhou, 730000 Gansu Province China; 4 Gansu key Laboratory of Molecular Diagnostics and Precision Medicine for Surgical Oncology, Gansu Provincial Hospital, Lanzhou, 730000 Gansu Province China; 5 Gansu Research Center of Prevention and Control Project for Digestive Oncology, Gansu Provincial Hospital, Lanzhou, 730000 Gansu Province China

**Keywords:** Pancreaticoduodenectomy, Pancreaticogastrostomy, Pancreaticojejunostomy, Meta-analysis, Pancreatic fistula, Delayed gastric emptying

## Abstract

**Background:**

To evaluate the outcomes of pancreaticogastrostomy and pancreaticojejunostomy after pancreatoduodenectomy with the help of a meta-analysis.

**Methods:**

Randomized controlled trials comparing pancreaticogastrostomy and pancreaticojejunostomy were searched electronically using PubMed, The Cochrane Library, and EMBASE. Fixed and random-effects were used to measure pooled estimates. Research indicators included pancreatic fistula, delayed gastric emptying, postoperative hemorrhage, intraperitoneal fluid collection, wound infection, overall postoperative complications, reoperation, and mortality.

**Results:**

Overall, 10 randomized controlled trials were included in this meta-analysis, with a total of 1629 patients. The overall incidences of pancreatic fistula and intra-abdominal collections were lower in the pancreaticogastrostomy group than in the pancreaticojejunostomy group (OR=0.73, 95% CI 0.55~0.96, *p*=0.02; OR=0.59, 95% CI 0.37~0.96, *p*=0.02, respectively). The incidence of B/C grade pancreatic fistula in the pancreaticogastrostomy group was lower than that in the pancreaticojejunostomy group, but no significant difference was observed (OR=0.61, 95%CI 0.34~1.09, *p*=0.09). Postoperative hemorrhage was more frequent in the pancreaticogastrostomy group than in the pancreaticojejunostomy group (OR=1.52; 95% CI 1.08~2.14, *p*=0.02). No significant differences in terms of delayed gastric emptying, wound infection, reoperation, overall postoperative complications, mortality, exocrine function, and hospital readmission were observed between groups.

**Conclusion:**

This meta-analysis suggests that pancreaticogastrostomy reduces the incidence of postoperative pancreatic fistula and intraperitoneal fluid collection but increases the risk of postoperative hemorrhage compared with pancreaticojejunostomy.

## Background

Pancreaticoduodenectomy (PD) (Table 1) is widely performed as the standard procedure for malignant and benign diseases of the pancreas and periampullary region. Given the improvements in surgical techniques and postoperative care, the perioperative mortality rate for PD has dropped to below 5% [1, 2]. However, the incidence of postoperative complications after PD is still as high as 30–50% [3] and mainly includes pancreatic fistula, delayed gastric emptying, and postoperative hemorrhage [4]. Postoperative pancreatic fistula is the most serious complication after PD, resulting in more hospital stages and charges, and directly affects patient prognosis [5]. To effectively reduce the incidence of postoperative pancreatic fistulas, methods of pancreatic outflow tract reconstruction are always the focus of exploration after PD. Pancreaticogastrostomy (PG) and pancreaticojejunostomy (PJ) are the two most common reconstruction methods after PD. A number of studies have reported the outcomes of these two reconstruction techniques to date, but the conclusions are inconsistent [6–9]. Further, previous systematic reviews and meta-analyses provide no adequate evidence to prove whether one of these methods is better than the other after PD. Given the recent increase in the number of publications of high-quality randomized controlled trials (RCTs) comparing the outcomes of PG and PJ, it is necessary to combine data from these RCTs for a new meta-analyses. Thus, in this study, we evaluate about approximately eight outcomes of PG and PJ after PD with the help of a meta-analysis of these RCTs.

**Table 1:** List of abbreviations

Full name	Abbreviations
Pancreaticoduodenectomy	PD
Pancreaticogastrostomy	PG
Pancreaticojejunostomy	PJ
Randomized controlled trials	RCTs
International Study Group on Pancreatic Fistula	ISGPF
Odds ratio	OR
Confidence interval	CI
Postoperative pancreatic fistula	POPF

## Methods

### Inclusion and exclusion criteria

The studies that met the following inclusion criteria were included in this meta-analysis: (1) the RCT compared PJ and PG anastomosis for pancreaticodigestive reconstruction after PD, (2) PD was performed in adult patients, and (3) Raw data was available for extraction. Further, the following studies were excluded: (1) studies that failed to meet any of the aforementioned points, (2) studies without data for retrieval, and (3) studies that did not report any postoperative complications related to our outcome.

### Outcome definitions

The primary outcome of this study was the incidence rate of pancreatic fistula. Most studies followed the International Study Group on Pancreatic Fistula (ISGPF) criteria for determining the rate ^21^; however, some studies used other definitions (Table 2). With regard to secondary outcomes, delayed gastric emptying was defined as the inability to return to a standard diet by the end of the first postoperative week or the need for nasogastric tube decompression for 10 days or more. Hemorrhage was characterized by intra-abdominal or digestive tract bleeding. Intraperitoneal fluid collection was defined as fluid collection with or without abscess. Overall postoperative complications were defined as the overall proportion of variety of complications observed in patients. Exocrine function was compared and evaluated through postoperative steatorrhea. Wound infection was defined as purulent and infected lesions at the surgical incision site. Reoperation rate was defined as percentage of postoperative complications that was resolved by surgical treatment. Mortality was defined as death during hospitalization or within 60 days after operation.

**Table 2:** The characteristics of 10 RCTs

ID	Years	Country	Study type	Cases PG/PJ	Mean age (years) PG/PJ	Female% PG/PJ	Operating time (h) PG/PJ	Hospital stay (days) PG/PJ	Definition of PF
Yeo et al.	1995	United States	Controlled randomized, single-center trial	73/72	62/62	40/34	7.4/7.2	17.1/17.7	Radiographically documented leak or >50 mL drainage of amylase-rich fluid on or after postoperative day 10.
Duffas et al.	2005	France	Single-blind, controlled randomized, multicenter trial	81/68	58/59	30/33	6.5/6.4	20.0/21.0	Fluid obtained through drains or percutaneous aspiration, containing at least 4 times normal serum values of amylase for 3 days, confirmed by fistulography, by upper gastrointestinal hydrosoluble contrast or enhanced Ct scan.
Bassi et al.	2005	Italy	Controlled randomized, single-center trial	69/82	59/56	25/31	5.6/5.9	14.2/15.4	Any clinical significant output of fluid, rich in amylase, confirmed by fistulography.
Fernandez-Cruz et al.	2008	Spain	Controlled randomized, single-center trial	53/55	63/63	24/17	5.0/5.2	12.0/16.0	ISGPF definition
Wellner UF et al.	2012	Germany	Controlled randomized, single-center trial	59/57	67/64	32/28	6.7/7.4	15.0/17.0	ISGPF definition
Topal et al.	2013	Belgium	Single blind, controlled randomized,multicenter trial	162/167	67/66	76/62	4.2/4.2	19.0/17.0	ISGPF definition
Figueras et al	2013	Spain	Single blind, controlled randomized, single-center trial	65/58	67/66	21/21	5.5/5.1	12.0/15.5	ISGPF definition
Grendar et al.	2014	Canada	Double blind, controlled randomized, single-center trial	48/50	64/68	28/21	5.8/5.9	17.4/14.0	Radiologically proven anastomotic leak or continued drainage (via drain, enterocutaneous fistula or wound) of lipase-rich fluid on postoperative day 10.
El Nakeeb et al.	2014	Egypt	Single-blind, controlled randomized, single-center trial	45/45	58/54	22/18	5.0/5.3	9.0/8.0	ISGPF definition
Keck et al.	2016	Germany	Single-blind, controlled randomized, multicenter trial	171/149	68/66	67/56	5.5/5.6	15.0/16.0	ISGPF definition

### Search strategy and data collection

This meta-analysis had been registered on PROSPERO, and the trial protocol number is CRD42021255642 (https://www.crd.york.ac.uk/prospero/). Full-text articles published between January 1995 and November 2019 were searched in PubMed, The Cochrane Library, and EMBASE. No limitations in terms of language of publication were applied. The following medical key words were used while searching: pancreaticoduodenectomy, pancreaticogastrostomy, and pancreaticojejunostomy. Besides, the references mentioned in the selected articles were also reviewed. Study selection and data extraction were performed by two reviewer independently based on the inclusion and exclusion criteria. Study reports included date on number of patients in each group, gender, mean age, definition of pancreatic fistula, reoperation, overall postoperative complications, mortality, exocrine function, and hospital readmission. Disagreements between reviewers were resolved by a discussion. The risk of bias graph and summary was used to analyze the quality of all RCTs.

### Statistical analyses

Meta-analysis was performed using the Cochrane Collaboration software (RevMan version 5.3). The Cochran Q test was performed, with a predefined significance threshold of 0.1, to estimate the presence of heterogeneity between studies. If no significant heterogeneity (*P*≥0.1) was observed, a fixed-effects model was used; otherwise, the heterogeneity and other reasons were further investigated or the random-effects model was used. The odds ratio (OR) with 95% confidence interval (CI) was calculated for dichotomous variables. Mean differences (MDs) were calculated for continuous variables. *P* < 0.05 was considered statistically significant.

## Results

**Fig. 1:**
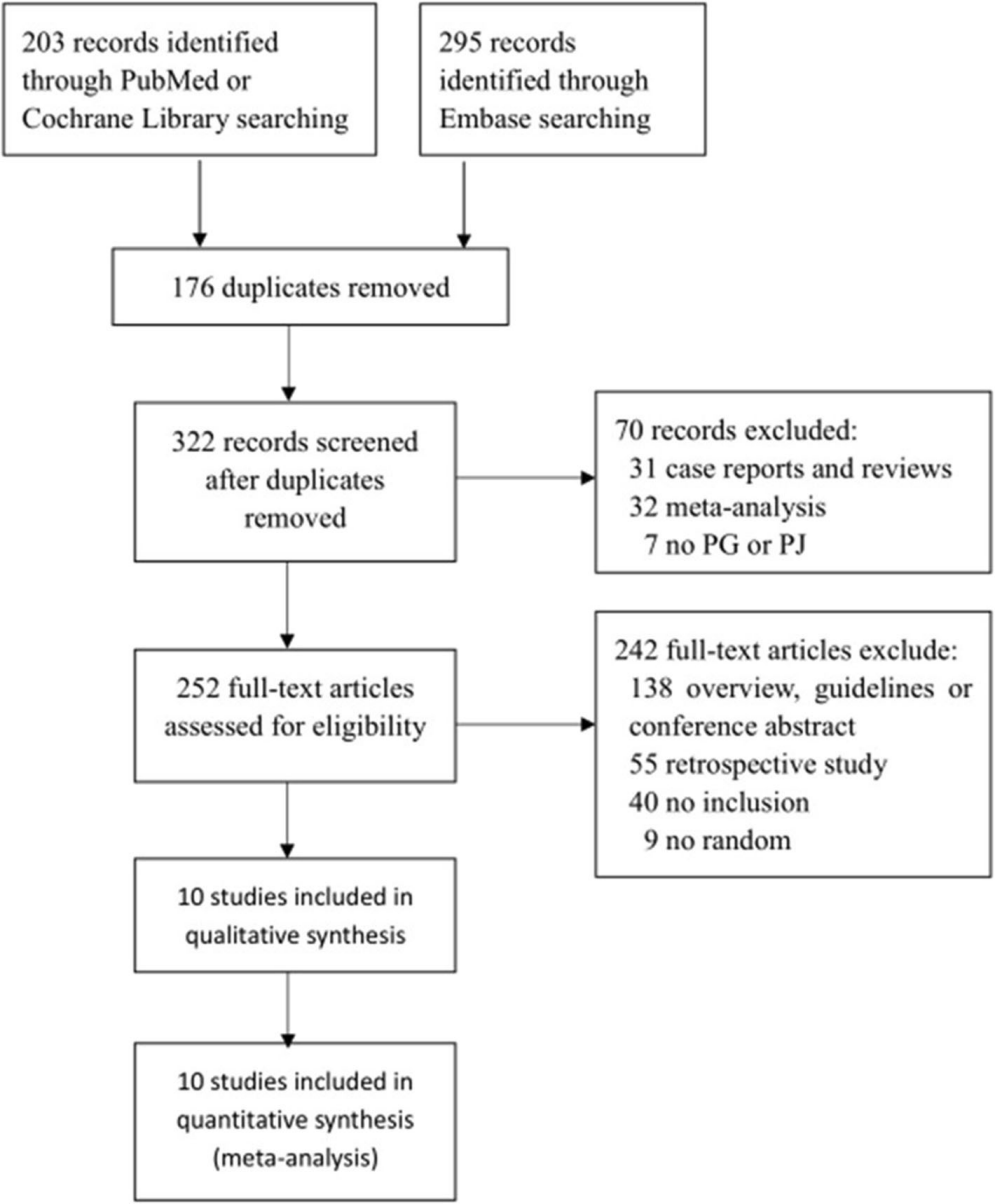
Study flow diagram

**Fig. 2:**
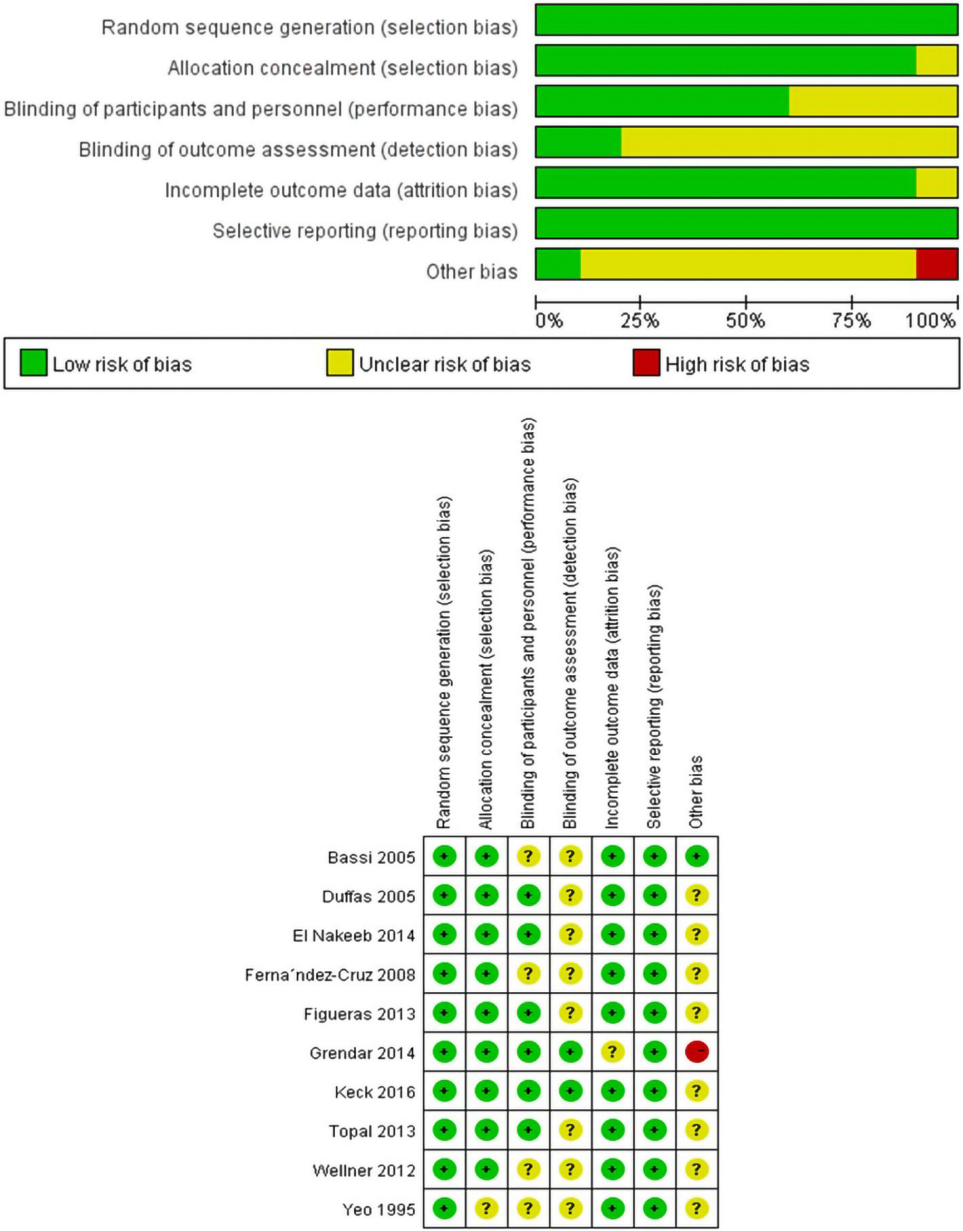
The quality of these 10 trials. The risk of bias graph and summary

In all, 10 randomized controlled trials published as full-text articles were included in the study based on the inclusion and exclusion criteria. These studies included 826 patients who underwent PG and 803 who underwent PJ after PD. We excluded 3 patients (2 from the PG group, 1 from the PJ group) from the total number of patients with perioperative in-house mortality (similar to the exclusion by Keck T et al. [10]), because of missing data. The specific patient selection process is shown in Fig. 1. The characteristics of the 10 included RCTs are shown in Table 1. The qualities of these 10 trials are shown in Fig. 2.

## Results of meta-analysis

### Pancreatic fistula

**Fig. 3:**
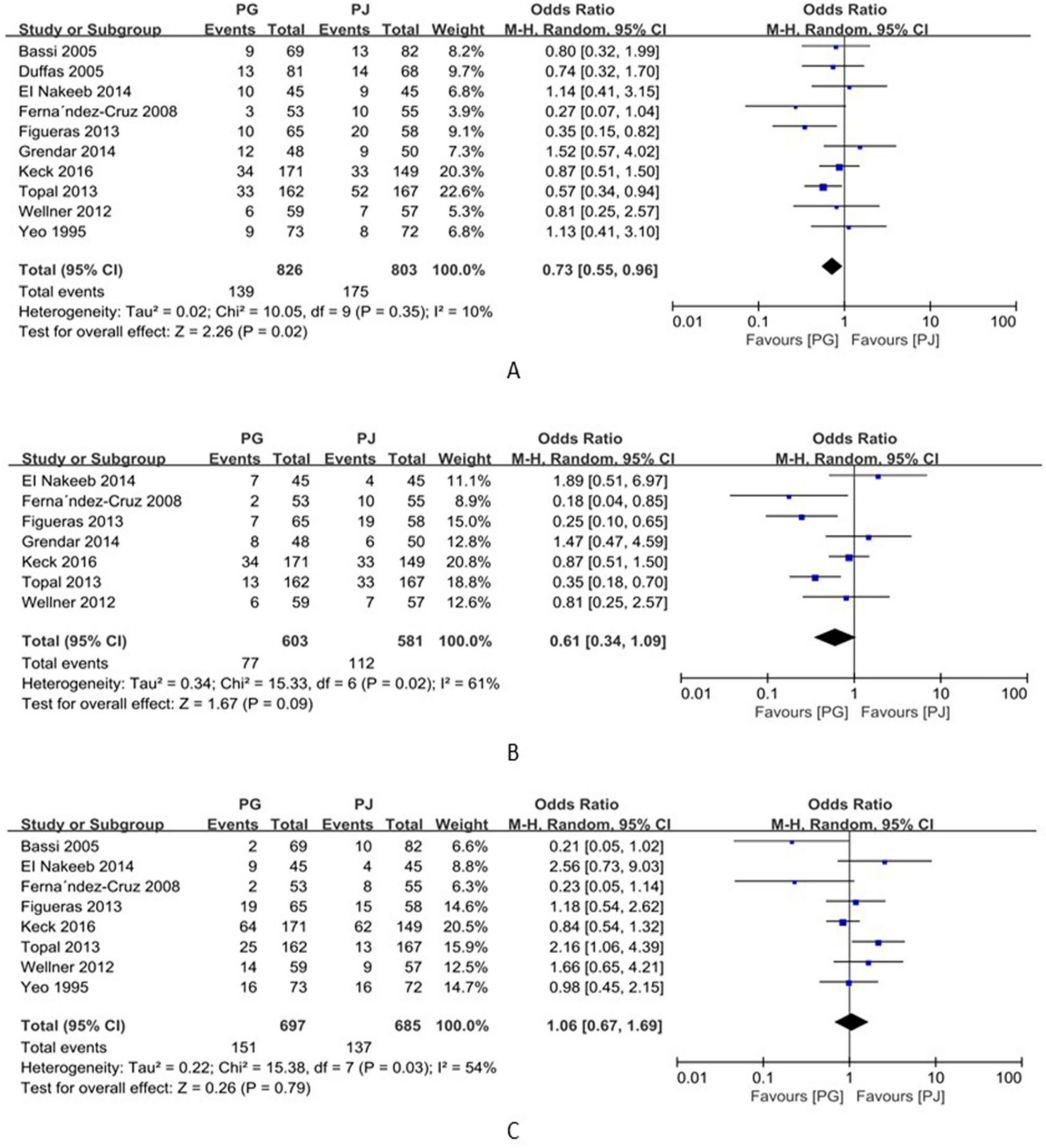
Forrest plot of meta-analysis: **A** postoperative pancreatic fistula, **B** the postoperative grade B/C pancreatic fistula, and **C** delayed gastric emptying; PD pancreaticogastrostomy; PJ pancreaticojejunostomy

Ten studies (including 1629 patients) reported postoperative pancreatic fistula (POPF) rates [10–19]. The pancreatic fistula rate was 16.8% (139/826) in the PG group and 21.8% (175/803) in the PJ group. Meta-analysis showed that the rate of occurrence of pancreatic fistula was significantly lower in the PG group than in the PJ group (OR = 0.73; 95% CI, 0.55~0.96; *p* = 0.02) (Fig. 3). Further, seven studies (including 1184 patients) reported pancreatic fistula (grade B/C) rates [10, 13–18]. The meta-analysis showed no significant difference in the incidence of pancreatic fistula (grade B/C) between the PG and PJ groups (OR=0.61; 95% CI, 0.34–1.09; *p*=0.09) (Fig. 3), but there was considerable statistical heterogeneity (*I*^*2*^=61%) (Fig. 3). As heterogeneity existed among these studies (*p*<0.1), a random-effects model was used to minimize the analytical error.

### Delayed gastric emptying

Eight studies including 1382 patients reported delayed gastric emptying [10, 11, 13–15, 17–19]. The incidence rates of delayed gastric emptying in the PG and PJ groups were 21.7% (151/697) and 20.0% (137/685), respectively. The meta-analysis showed no significant difference in delayed gastric emptying between the PG and PJ groups (OR=1.06; 95% CI, 0.67–1.69; *p*=0.79) (Fig. 3). However, there was considerable statistical heterogeneity (*I*^*2*^=54%) (Fig. 3). As heterogeneity existed among these studies (*p*<0.1), a random-effects model was used to minimize the analytical error.

### Postoperative hemorrhage

**Fig. 4:**
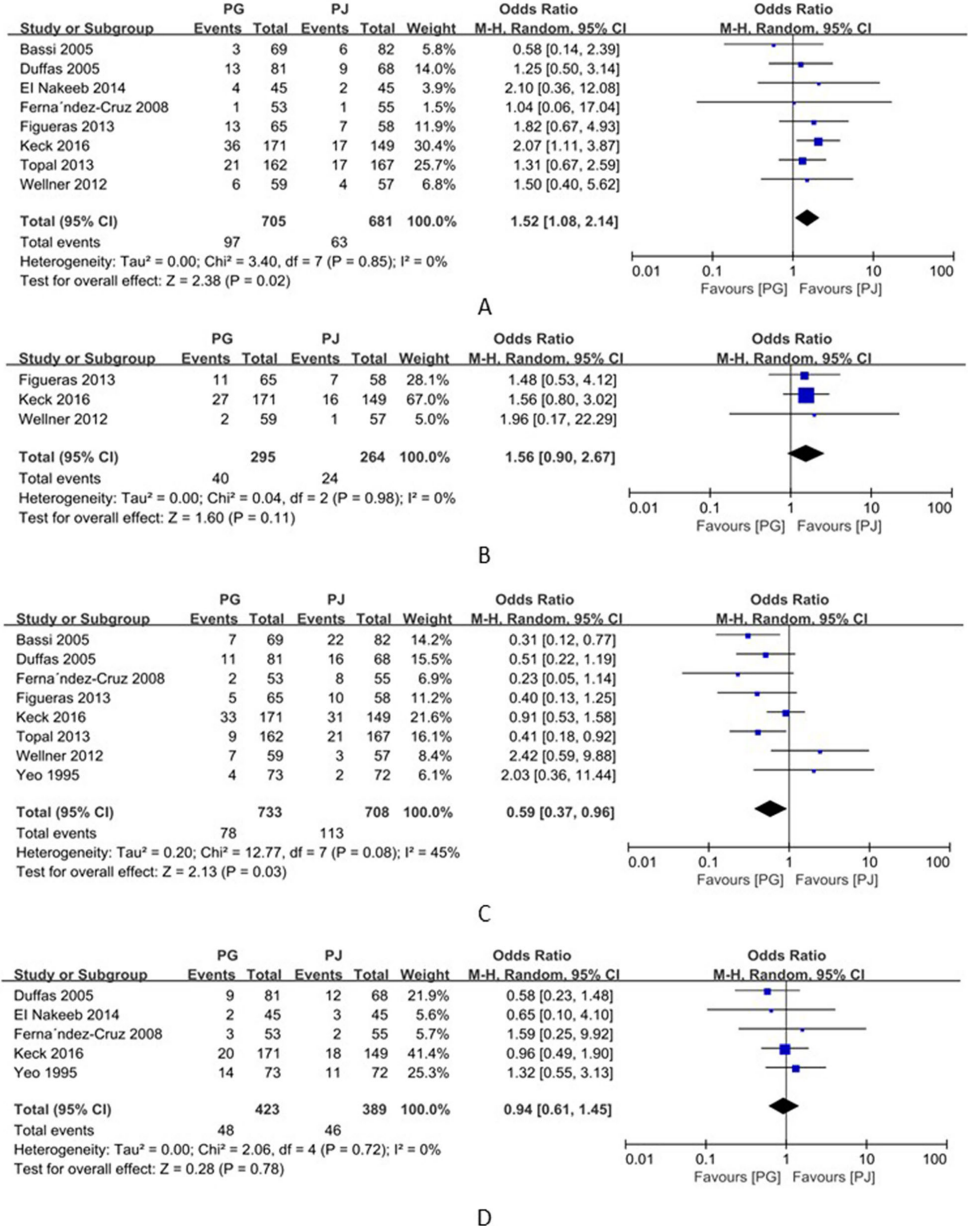
Forrest plot of meta-analysis: **A** postoperative hemorrhage, **B** postoperative grade B/C hemorrhage, **C** postoperative intraperitoneal fluid collection, and **D** postoperative wound inflection; PD pancreaticogastrostomy; PJ pancreaticojejunostomy

Eight studies including 1386 patients reported postoperative hemorrhage as a complication [10–15, 17, 18]. The incidence rates of postoperative hemorrhage in the PG and PJ groups was 13.8% (97/705) and 9.25% (63/681), respectively. The meta-analysis showed that the incidence rate of hemorrhage was significantly lower in the PJ group than in the PG group (OR=1.52; 95% CI, 1.08–2.14; *p*=0.02) (Fig. 4). Further, three studies reported the incidence of postoperative hemorrhage (grade B/C) [10, 15, 18] and the combined analysis of these studies showed no significant difference between PG and PJ groups (OR=1.56; 95% CI, 0.90–2.67; *p*=0.11) (Fig. 4).

### Intraperitoneal fluid collection

Eight studies including 1441 patients reported results on intraperitoneal fluid collection [10–12, 14, 15, 17–19]. The rates of intraperitoneal fluid collection in the PG and PJ groups were 10.7% (78/733) and 16.0% (113/708), respectively. This meta-analysis showed that the rate of intraperitoneal fluid collection was significant lower in the PG group than in the PJ group (OR=0.59; 95% CI, 0.37–0.96; *p*=0.03) (Fig. 4), and there was considerable statistical heterogeneity (*I*^*2*^=45%) (Fig. 4). As heterogeneity existed among these studies (*p*<0.1), a random-effects model was used to minimize the analytical error.

### Wound infection

In all, five studies including 812 patients reported on wound infection in patients [10, 12–14, 19]. The rates of infection in the PG and PJ groups were 11.4% (48/423) and 11.8% (46/389), respectively. Accordingly, the meta-analysis showed no significant difference in wound infection between the PG and PJ groups (OR=0.94; 95% CI, 0.61–1.45; *p* = 0.78) (Fig. 4).

### Overall postoperative complications

**Fig. 5:**
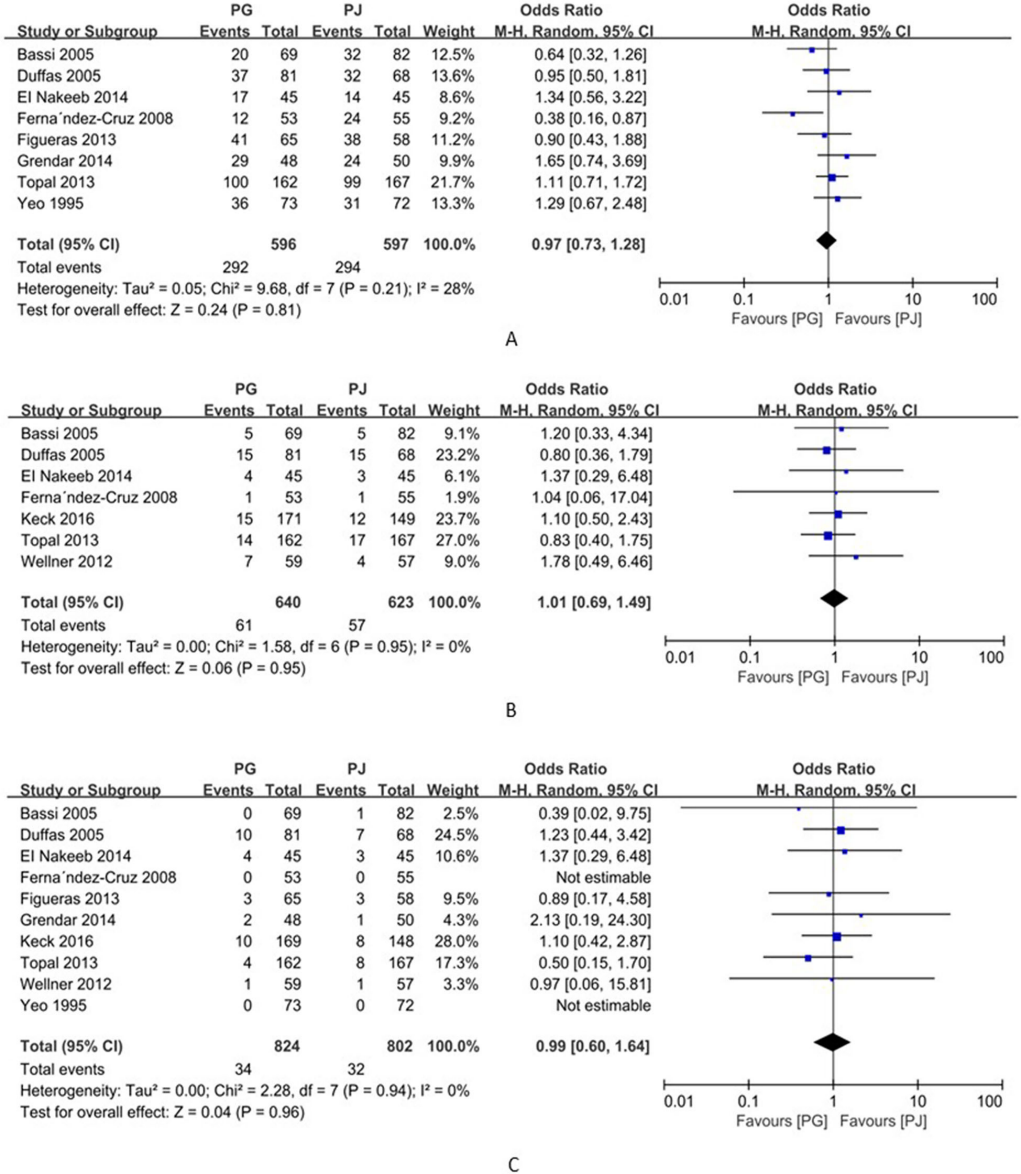
Forrest plot of meta-analysis: **A** overall rate of postoperative complications, **B** postoperative reoperation, and **C** postoperative mortality; PD pancreaticogastrostomy; PJ pancreaticojejunostomy

Overall postoperative complications included intra-abdominal and medical complications. Eight studies (including 1193 patients) reported the rates of overall postoperative complications in patients [11–17, 19]. The overall postoperative complications rate was 49.0% (292/596) in the PG group and 49.3% (294/597) in the PJ group. Accordingly, the meta-analysis showed no significant difference in the overall postoperative complications rate between groups (OR=0.97; 95% CI, 0.73–1.28; *p*=0.81) (Fig. 5).

### Reoperation rate

Reasons for reoperation mainly included the following: continuous postoperative bleeding (grade B/C) not suitable for conservative treatment, pancreatic fistula (grade B/C) not suitable for percutaneous drainage, and anastomotic dehiscence or obstruction. Seven studies including 1263 patients reported reoperations [10–14, 17, 18]. The reoperation rates were 9.53% (61/640) and 9.15% (57/623) in the PG and PJ groups, respectively, with no significant difference observed between groups (OR=1.01; 95% CI, 0.61–1.49; *p*=0.95) (Fig. 5).

### Mortality

Ten studies (including 1626 patients) reported on the incidence of postoperative mortality rates [10–19]. The overall mortality rates were 4.1% (34/824) and 4.0% (32/802) in the PG and PJ groups, respectively, with no significant difference between groups (OR=0.99; 95% CI, 0.60–1.64; *p*=0.96) (Fig. 5).

### Exocrine function

**Fig. 6:**
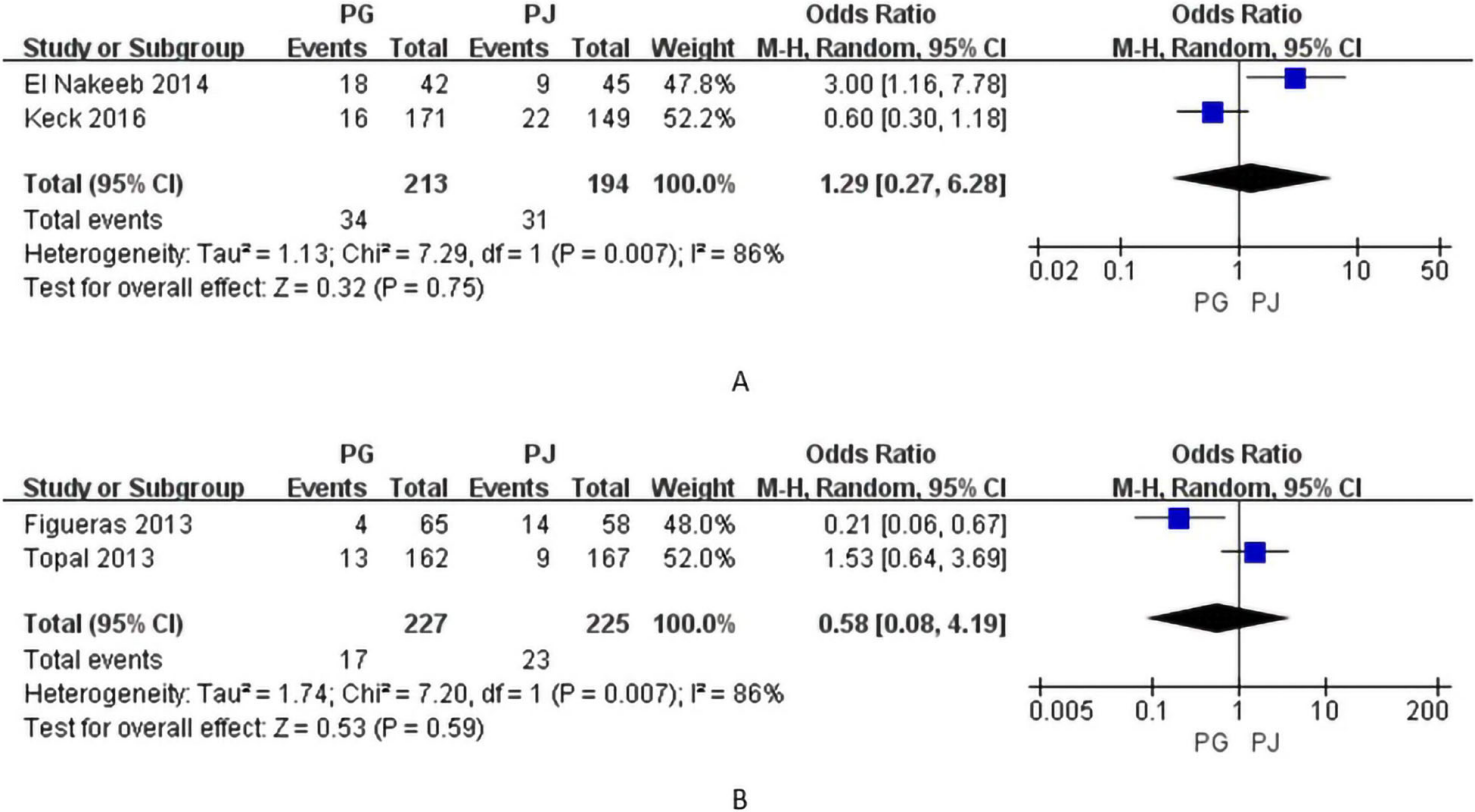
Forrest plot of meta-analysis: **A** postoperative exocrine function, and **B** postoperative hospital readmission; PD pancreaticogastrostomy; PJ pancreaticojejunostomy

Three studies compared the results of postoperative exocrine function comparison [10, 13, 15]. However, one study compared exocrine function by stool elastase [15] and two studies compared exocrine function by steatorrhea [10, 13]. Accordingly, we compared exocrine function through postoperative steatorrhea. The postoperative steatorrhea rates were 15.96% (34/213) and 15.97% (31/194) in the PG and PJ groups, respectively, with no significant difference between groups (OR=1.29; 95% CI, 0.27–6.28; p=0.75) (Fig. 6).

### Hospital readmission

Two studies reported the results of hospital readmission [15, 17]. Hospital readmission rates were 7.5% (17/227) and 10.2% (23/225) in the PG and PJ groups, respectively. The hospital readmission rate is slightly lower in PG group than PJ group, but there was no significant difference between groups (OR=0.58; 95% CI, 0.08–4.19; p=0.59) (Fig. 6).

## Discussion

Pancreatic fistula is one of the most frequent complications of PD that occurs when pancreatic anastomosis fails to heal following surgery [20]. Depending on the clinical impact, ISGPF has established the grading of pancreatic fistula (grades A, B, and C) [21] depending on the clinical impact, wherein grade B/C pancreatic fistula is associated with a deterioration in the clinical status of patients, necessitating intensive care management, and may lead to delayed recovery, reoperation risk, and a distinct risk of mortality. Patient factors, including age, preoperative biliary drainage, body mass index, vital organ function, and frail or not, also affect the incidence of POPF [22–25], but the treatment of the pancreatic stump and the method of anastomosis are still key to prevent the occurrence of pancreatic fistula. Nevertheless, pancreatic outflow tract reconstruction after PD remains to be a challenging problem after PD to date, even for experienced surgeons, and based on published literature, no single reconstruction method is suitable for all patients. Some retrospective studies have reported that the incidence of POPF in the PG group was lower than that in the PJ group [26–28], whereas other original studies and meta-analyses show that PG does not reduce the incidence of POPF [11, 15, 29, 30]. In this meta-analysis, we showed that the PG group was associated with a lower incidence of pancreatic fistula than the PJ group. However, the incidence rates of grade B/C pancreatic fistula in the PG and PJ groups were 12.8% (77/603) and 19.3% (112/581), respectively; although the difference was not statistically significant between the two groups, the incidence rate in the former was considerably lower than that in the latter group. This is consistent with the outcomes reported in a recent meta-analysis [31]. The lower incidence rate of pancreatic fistula after PG may be associated with the following factors: high blood supply to the stomach is helpful for the healing of the anastomosis, the stomach acids can inhibit trypsin activation and bacterial breeding, and the gastric wall is helpful for the fixation of residual pancreas. Moreover, a French study showed that the incidence rate of POPF in patients with a soft pancreatic texture was significantly higher than the rate in patients with a hard pancreatic texture or those with the presence of fibrosis [32], This is in line with the conclusion reported by a Japanese study reporting that a small pancreatic duct (≤3 mm) or soft pancreas have the greatest risk of developing a pancreatic fistula [33]. However, the proportion of soft textured pancreas between patients in both groups, PG and PJ, was comparable in the studies included in our meta-analysis. Therefore, PG showed superiority in reducing the incidence of pancreatic fistula, compared with PJ. Finally, heterogeneity in the occurrence of grade B/C pancreatic fistula (*I*^*2*^= 61%) may have resulted from of the use of different definitions among the included studies as the incidence of pancreatic fistula depends on and varies with different definitions [34].

Delayed gastric emptying is another complication after PD; it was reported by Warshaw in 1985 [35], and since then, the risk factors of delayed gastric emptying have been studied. Some previous studies have reported that low motilin levels [36–38], vagus nerve injury, pylorospasm ischemia, pancreatic fistula, and mechanical factors are associated with delayed gastric emptying. Meanwhile, the effects of different anastomosis methods on delayed gastric emptying have also been studied. Our meta-analysis showed no significant difference in delayed gastric emptying between the PG and PJ groups. This was consistent with the findings by Park et al. [39]. Moreover, Figueras et al. found that the incidence rates of grade C delayed gastric emptying in the PG and PJ groups were 9% (6/65) and 7% (4/58), respectively, with no significant difference between groups [15]. However, there was considerable statistical heterogeneity in the result of delayed gastric emptying (*I*^*2*^= 53%); this may be because the proportion of patients who underwent pylorus-preserving pancreaticoduodenectomy with gastric partition varied among the included studies and some studies showed that the incidence of delayed gastric emptying was higher in the pylorus-preserving pancreaticoduodenectomy with gastric partition group than in the PD group [40, 41].

With regard to postoperative hemorrhage, a Japanese study showed that the rate of bleeding was similar between PG and PJ groups [42]. This meta-analysis showed that the hemorrhage rate was higher in the PG group than in the PJ group, but grade B/C hemorrhage rate was not different between PG and PJ groups; this may be one of the reasons why the rate of reoperation and mortality was not different between the two groups, because patients with grade B and C postoperative hemorrhage had significantly higher postoperative postoperative complications compared with those without postoperative hemorrhage or those with grade A postoperative hemorrhage based on the (International Study Group of Pancreatic Surgery) definition and clinical grading of hemorrhage [43]. The other possible explanation for this finding is that the alkaline pancreatic juice is emptied directly into the stomach in PG, which results in alkaline gastritis, reflux esophagitis, gastrointestinal dysfunction, and increased incidence of stress ulcer. Moreover, the pancreatogastric anastomosis is exposed to gastric secretions. These factors may lead to a high hemorrhage rate in the PG group.

Lower intra-abdominal fluid collection rate was observed in the PG group compared with the PJ group, which may be associated with the low incidence of pancreatic fistula in the PG group given that pancreatic fistulas are associated with intra-abdominal fluid collection [44]. Reoperation rate was not different between PG and PJ groups because an increasing number of cases of pancreatic fistulas and hemorrhages can be successfully managed by conservative treatment. No significant difference was found in the overall postoperative complications and mortality rates between the two groups. Apart from the abovementioned factors, intraoperative blood loss, postoperative care and nutritional status, use of prophylactic somatostatin and residual pancreatic secretion, and surgeon-related factors, can affect the incidence of overall postoperative complications and mortality after PD.

Three studies included in this meta-analysis evaluated endocrine and exocrine functions and found no differences in terms of endocrine function by comparing the prevalence of diabetes mellitus between PG and PJ [10, 13, 15]. This finding was consistent with the results reported by Schmidt et al. [45]. With regard to residual pancreatic exocrine function after PD, some retrospective studies have reported that patients who underwent PD with PG presented with considerably higher pancreatic exocrine insufficiency than patients who underwent PD with PJ [46, 47]. Similarly, El Nakeeb et al. found a higher incidence of steatorrhea 1 year after PG [13]. However, Figueras et al. found high levels of stool elastase 3–6 months after PG [15], suggesting better exocrine function in the PG group; this is in line with the findings reported by Keck et al. [10]. However, this is still controversial; therefore, high-quality RCTs comparing PG and PJ with a consistent biochemical index and long-term outcomes are still required to analyze the relationship between exocrine function and type of anastomosis and the factors affecting the exocrine function.

The limitations of meta-analysis include the following: the number of included studies was limited, the definition of pancreatic fistula was not unified among all published studies, and different surgical methods (PD and PPPD) were used. These factors may affect the reliability of the conclusion. In addition, the current study was not registered and may also have been biased in its smallness, but we still followed strict systematic review procedures.

## Conclusion

This study shows that PG reduces the incidence of postoperative pancreatic fistula and intraperitoneal fluid collection, but does not reduce the risk of delayed gastric emptying, postoperative reoperation rate, wound infection, overall postoperative complications, exocrine function, hospital readmission, and mortality rates. Further, PG increases the incidence of postoperative bleeding. Thus, PG cannot be considered superior to PJ. At the same time, a variety of improved anastomotic methods are also gradually applied in clinical practice, which include modified invaginated PJ and modified duct-to-mucosa PJ among others [48, 49]. Accordingly, further high-quality RCTs are still required for the validation of these results is necessary.

## Data Availability

Full-text articles published were searched in PubMed (https://pubmed.ncbi.nlm.nih.gov/), The Cochrane Library (https://www.cochranelibrary.com), and EMBASE (https://www.embase.com), and figures have made by the Cochrane Collaboration software RevMan version 5.3. So data sharing is not applicable to this article as no datasets were generated or analyzed during the current study.
